# On the razor's edge: navigating mature experience and the challenges of aging in modern surgery

**DOI:** 10.3389/fsurg.2024.1383531

**Published:** 2024-03-12

**Authors:** Luca Bertolaccini, Monica Casiraghi, Lorenzo Spaggiari

**Affiliations:** ^1^ Department of Thoracic Surgery, IEO, European Institute of Oncology IRCCS, Milan, Italy; ^2^ Department of Oncology and Hemato-Oncology, University of Milan, Milan, Italy

**Keywords:** lung cancer, surgeon, ageing, mental impact, physical evaluation

The surgical workforce is ageing, with nearly a third of surgeons over 55 years old currently in practice. This demographic shift requires careful attention. On the one hand, clinical studies have shown that patients treated by surgeons over 65 demonstrate a 7% reduction in the likelihood of adverse outcomes, with a nearly linear relationship between the surgeon's age and a decrease in complication and postoperative mortality rates (1). On the other hand, 50% of healthcare professionals have reported burnout episodes more than once a year, significantly associated with age under 40 (i.e., fewer years as a specialist), fear of legal issues, unhealthy lifestyle, abstaining from recreational activities, depersonalisation during patient care, fatigue at the end of the day, and job dissatisfaction (2).

Therefore, physicians' decisions to retire from clinical practice are influenced by various factors such as low job satisfaction, medical-legal issues with increasing professional liability expenses, and, not least, insufficient financial remuneration. Conversely, those who postpone retirement often do so for professional satisfaction, institutional flexibility, a sense of responsibility toward patients, and a desire to remain active. Some countries have mandatory retirement ages for physicians, such as India (65 years for public sector doctors), China and Russia (60 years for male doctors, 55 years for females), and Pakistan (70 years). However, these mandatory age limits have been revoked in some contexts to address physician shortages, as seen in Germany and the United Kingdom. There are no national compulsory retirement ages for physicians in the United States and many other countries (3).

In Italy, the retirement age for surgeons is 67 years, regardless of their psychophysical abilities. A notable observation is that many surgeons, once they exit the public healthcare system, choose to practice in the private sector, especially in private healthcare. This transition results in the loss of experience for the public sector, which has trained them, and a significant reduction in the availability of teaching (4). The need for more medical personnel in many Italian hospitals poses a considerable challenge to the national healthcare system. This issue is further compounded by Italy having the highest number of elderly surgeons in Europe, with a median age of 57, indicating a potential worsening of the situation in the next 5–10 years. A more critical problem lies in the varying attractiveness of different medical specialisations. Specialisations offering greater earning potential in the private sector and greater flexibility in working hours quickly fill the open positions. In contrast, other critical fields, such as surgery, where there is a greater need for personnel, need help attracting candidates. This creates a crucial imbalance in the distribution of specialised medical professionals.

Considering the time required for medical training, an immediate solution to the physician shortage is not feasible. However, measures can be taken to alleviate this situation partially. Like the rest of the population, surgeons experience age-related declines in neurocognitive, sensory, and neuromuscular functions, with potential impacts on the quality of patient care. The eloquent metaphor of greying hair and reduced elasticity, forcing a man to open rather than jump over a gate, captures the essence of this transition. Ferdinand Sauerbruch, a surgery giant in his time, embodies the narrative of a once-brilliant surgeon whose late career was marked by cognitive decline (5).

Recognising variability in cognitive function among individuals and rejecting a one-size-fits-all solution, attention shifts toward assessing functional age rather than imposing an arbitrary retirement age. The limitations of existing methods for evaluating older surgeons underscore the need for a structured program: a comprehensive, multidisciplinary, objective, and confidential assessment (5). The American College of Surgeons has tackled the challenge of ageing surgeons by recommending objective evaluations as part of the credentialing renewal process. These evaluations, conducted through peer-reviewed methods, include practice assessment, chart review, decision review, patient feedback, and video review of surgical procedures with mentoring from younger surgeons. Additionally, the American College of Surgeons suggests a comprehensive program that also incorporates neuropsychological assessment, such as the MicroCog test, which measures neurocognitive function, assessing reaction time, numeric recall, verbal memory, spatial-visual acuity, and mental calculation (all elements that tend to decline with age).

Extending physicians' careers beyond 70 presents significant pros and cons, and its implementation should be voluntary, respecting individual choices without imposing directive commitments. Stepping away from administrative roles to focus on patient care and practical teaching can be a wise decision. Experienced surgeons can provide high-quality patient care and serve as valuable mentors and teachers for younger surgeons. Among the advantages of allowing physicians to continue practising beyond 70 is the accumulation of experience and skills. Older professionals can offer a unique perspective, resulting from decades of experience, contributing to greater clinical wisdom and knowledge transmission to younger colleagues. This continuity can ensure stability and a gradual transition of responsibilities. On the other hand, a generational disparity in medical practice may emerge.

The introduction of new technologies, such as surgical robots, can play a crucial role in reducing the age-related gap in medical practice, offering significant benefits derived from the consolidated experience of older physicians. This integration can be a promising perspective for the future of surgery and medical training in general. Surgical robots, with millimetre precision and advanced capabilities, can aid older physicians, compensating for any physical limitations related to age. Furthermore, it is worth noting the emerging role of additional technologies, such as artificial intelligence, which could play a pivotal role in further facilitating the integration of advanced tools to support ageing surgeons in the operating room. AI holds significant potential in assisting surgeons with tasks ranging from preoperative planning to real-time decision-making during procedures. By harnessing the power of artificial intelligence alongside surgical robots and other advanced technologies, healthcare professionals can enhance the capabilities of senior surgeons, ultimately improving patient outcomes while alleviating some of the challenges associated with the ageing surgical workforce.

The voluntary approach is crucial to respecting the freedom of choice for older physicians. Allowing them to extend their careers based on their physical and psychological conditions can contribute to maintaining a more flexible and sustainable working environment. However, it is essential to ensure that this choice is informed and supported by capacity assessment programs to preserve patient safety and the quality of medical care. Surgeons over 70 years old should follow standards similar to those used for the annual recertification of pilots, including medical evaluation, psychometric tests, breathalyser testing, and screening for any narcotics in the urine (6). It is crucial to emphasise that psychophysical evaluation should be a generalised practice for all physicians, with a decreasing interval as age increases. This approach would ensure continuous and tailored monitoring of the specific needs of healthcare professionals throughout their careers. Initially, psychophysical evaluation should be applied to all physicians, regardless of age, to identify early signs of cognitive and physical decline. As physicians age, the interval between assessments could be reduced, allowing closer monitoring and timely identification of changes in their ability to practice safely and effectively. This practice would contribute to ensuring patient safety and preserving healthcare quality. Additionally, it would allow physicians to be aware of their changes and make informed decisions about the duration of their professional careers.

In conclusion, managing the ageing of the surgical workforce requires a balanced approach that recognises the diversity of individual capabilities and promotes sustainable working conditions. The integration of advanced technologies, such as surgical robots, can revolutionise medical practice, allowing older physicians to continue making significant contributions. This synergy between human experience and technological innovation is an exciting prospect for the future, where innovation contributes to bridging the generational gap. The medical community, healthcare institutions, and regulatory bodies must collaborate to develop policies and programs that preserve the quality of patient care while ensuring the well-being and satisfaction of surgeons of all ages.
